# The Mediating Effect of Psychological Ownership on the Relationship between Value Co-Creation and the In-App Purchasing Intention of Mobile Games Players

**DOI:** 10.3390/bs13030205

**Published:** 2023-02-27

**Authors:** Yiwen Li, Jaewoo Joo

**Affiliations:** Department of Marketing, College of Business Administration, Kookmin University, Seoul 02707, Republic of Korea

**Keywords:** value co-creation, psychological ownership, in-app purchasing, mobile game

## Abstract

In previous research on in-app purchasing, one of the revenue sources for mobile games focuses on users’ unilateral relationships, such as their achievement, loyalty, and perception. However, little has been discussed about the commercial impact of the bilateral relationship. We extend discussions by examining an unprecedented issue, that is, the role of the bilateral relationships between users and mobile game companies in increasing in-app purchasing intention. We borrow from the business literature and psychology to hypothesize that when mobile game users co-create value with a mobile game company, their psychological ownership of the mobile game increases, which in turn increases their in-app purchasing intentions. To test this hypothesis, we conducted a carefully designed study by recruiting eighty-six Chinese game users. Half of the participants were exposed to an imaginary mobile game whose interface allowed them to co-create value with the mobile game company and the other half were exposed to an identical mobile game whose interface did not. We recruited participants from the two online platforms in which Chinese mobile game players gather—Weibo and WeChat Moment. Using SPSS 26, we conducted an independent samples test to test the effect of value co-creation and employed Hayes Model 4 to test whether psychological ownership mediated the relationship between value co-creation and in-app purchasing intention. We found that (1) when participants were allowed to co-create value, their in-app purchasing intentions increased, and (2) the relationship between value co-creation and in-app purchasing intention was mediated by psychological ownership. Our findings provide fresh insights for mobile game designers and marketers.

## 1. Introduction

Mobile game companies now search for a revenue source. The mobile game industry experienced explosive growth during the COVID-19 lockdowns because playing mobile games became the most popular indoor activity [1,2]. As numerous newly developed games were quickly introduced, market competition became fierce. For instance, the annual revenue of a Chinese mobile game, Honor of Kings, increased from 0.44 billion USD in 2016 to 1.05 billion USD in 2017 and then stagnated in the next two years. Then, this number increased to 1.51 billion USD in 2020 and then suddenly dropped to 1.03 billion USD in 2021 [3].

Since enhanced market competition decreased the revenue of mobile game companies, they actively searched for alternative revenue sources. According to prior research, their revenue sources include charging for downloaded applications [4], advertising in mobile games [5], and users’ in-app purchasing [6]. Among the three revenue sources, users’ in-app purchasing has been less discussed. In-app purchasing, or the behavior that mobile game users spend their money on buying virtual currency and mobile items, is found to be determined by users’ unilateral relationships with a mobile game. These relationships include, for instance, users’ achievements in a mobile game [7], their loyalty and addiction toward a mobile game [8], or their perception of the game items in a mobile game [9]. Although previous research on in-app purchasing highlighted unilateral relationships [10,11,12,13], it did not determine the bilateral relationships between users and a mobile game company. That means that in-app purchasing is not merely governed by the strategies or interfaces determined by a mobile game company but is also influenced by the relationship between the users and the mobile game the users play. Research on this topic is scant; however, mobile game companies often seek users when developing or introducing new games and also try to interact with them through online game communities.

Therefore, firstly, we aim to examine whether the bilateral relationships between users and a mobile game company increase users’ in-app purchasing intention. To achieve this goal, we borrow insights from the business literature on the topic of value co-creation. It shows that as the market transforms from production-centered to consumer-centered, value co-creation activities and their commercial effects attract attention in multiple industries, including tourism [10], hotels [11], sports [12], and online retail stores [13]. Now, researchers generally reach a consensus that value co-creation enhances the perceived value of products, consumer satisfaction, and finally, a company’s reputation [10,11,12,13]. 

Secondly, we also aim to demystify the relationship between value co-creation and in-app purchasing intention by proposing an established concept called psychological ownership. According to numerous previous studies, this “feeling” of mine could be an asset for companies because it increased consumers’ purchase intention of a product. This is because when they invest time and resources into a company, they are cognitively and emotionally connected to the company and its products [14]. Psychological ownership is particularly important in the game industry because game users are not allowed to physically possess or permanently access virtual currency or game items. Instead, they “feel” they own currency or items [15].

In summary, we propose that when mobile game users co-create value with a mobile game company, their psychological ownership of the mobile game increases, which in turn increases their in-app purchasing intentions. In the following sections, we review the literature on value co-creation, in-app purchasing intention, and psychological ownership. Then, we introduce a carefully designed study and report the obtained findings. Finally, we discuss findings, contributions, limitations, and future research. 

## 2. Literature Review

### 2.1. Value Co-Creation

Value co-creation is an activity in which consumers and a company create value together [14]. More specifically, consumers actively participate in an experience environment that a company creates and invest their time and resources to build a personalized experience, creating value collaboratively [15,16].

The commercial impact of value co-creation has been frequently reported. For instance, when a sports center increased interactions with visitors, visitor satisfaction with the sports center increased [12]. When consumers participated in a brand value co-creation, their purchase intention of the same brand increased [16]. Contemporary researchers go beyond merely demonstrating its impact; some propose its process model, others introduce its organizational version [17], and others clarify and break down the concept of value co-creation in more detail [18].

Although research about value co-creation is abundant, its commercial impact in the mobile game context has not been discussed. Since value co-creation was considered an important contributor to the service industry, it needs to be discussed in the gaming industry.

### 2.2. In-App Purchasing Intention

In-app purchasing intention measures how much a game user is willing to spend their money on buying virtual currency [5]. Virtual currency can be exchanged with other in-app products, including the skins of a character or the items used in the mobile game. Since purchasing intention is an indicator of actual purchase, in-app purchasing intention can be considered as users’ actual willingness to be involved with a mobile game [19].

We propose that when mobile game users are allowed to interact with a mobile game company, their in-app purchasing intention increases. Similar findings have already been obtained from a study within the context of Social Network Services (SNS) in that value co-creation of an SNS fan page increased fans’ purchasing intention of the SNS [20,21].

**H1.**  *Value co-creation will increase in-app purchasing intention.*

### 2.3. Psychological Ownership

Psychological ownership is a feeling of mine [22]. The core of psychological ownership is the feeling of possessiveness and the spiritual tie with a product [23]. It is a form of emotional attachment that a consumer has with his or her own products [24]. Psychological ownership does not require legal ownership [25]. A well-known example of psychological ownership is the IKEA effect. Research on this topic shows that participants who created origami value it as much as the other origami created by a professional expert. That means people develop an emotional attachment to a product when developing and owning it psychologically [26].

Prior research shows that psychological ownership plays a significant role in consumer purchase intention. For instance, social media users who have a high degree of psychological ownership indicate a greater purchasing intention [27]. When consumers develop psychological ownership of a product, they invest time and resources into it and then feel cognitively and emotionally connected to the product [28]. Sometimes, when a company satisfies consumer needs by developing a product, consumers sacrifice their economic interest and invest their effort to own the product psychologically [29].

We believe a similar effect will be observed in the mobile industry. When game users actively interact with a mobile game company, their psychological ownership of the mobile game increases, which in turn increases their intention to buy virtual currency in the mobile game.

**H2.**  *Psychological ownership will mediate the relationship between value co-creation and in-app purchasing intention.*

**Figure 1:**
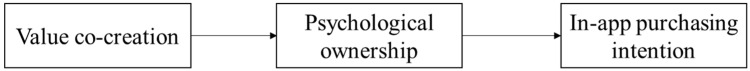
Research framework.

In summary, the research framework of our study is as follows (see Figure 1).

## 3. Study

### 3.1. Participants

We tested our two hypotheses by generating a hypothetical mobile game and recruiting real game users. We recruited participants in China because China is one of the most lucrative gaming markets in the world. According to Newzoo.com, Chinese game revenue is estimated to be the largest in the world at $US 46 billion in 2021 and the number of game players in China (744.1 million) is three times greater than the number of game players in the US (209.8 million). We paid extra effort to recruit the right participants. First, we avoided children because, since October 2019, Chinese children have not been legally allowed to play mobile games anymore. According to the notification about preventing minors from indulging in online games, as announced by the National Press and Publication Administration of China [30], minors, including students and children are restrained from playing games. Second, we also avoided adults who had no mobile game experience in the past. This is because non-game users have a limited understanding of in-app purchasing behavior. As such, we recruited participants from the two online platforms in which Chinese mobile game players gather: Weibo and WeChat Moment. We recruited a total of 86 participants from 2 May 2022 to 10 May 2022. Among them, 43% were male (57% female) and their age distribution was between 18 and 32 (M_age_ = 24.05, SD_age_ = 2.86). 

### 3.2. Experiment Stimuli

We created a hypothetical mobile game by changing a famous Chinese mobile game called Honor of Kings. Note that this game is the first free game application and a multiplayer online battle arena (MOBA) mobile game, having more than 100 million daily active users in China [31].

### 3.3. Experiment Design

We employed a 2 (Value co-creation: No vs. Yes) between-subjects design. To manipulate value co-creation, we manipulated the interface in which three specific features are different. First, the location of the customer service button was manipulated so that participants can find customer service easily (vs. difficult). Second, the information about the game balance optimization was manipulated so that participants could give feedback about it (vs. not). Finally, the information about the new skin design was manipulated so that participants could give feedback by entering scores (vs. not). To sum up, in the no-value co-creation condition, participants were not allowed to interact with the mobile game company. In contrast, in the value co-creation condition, participants find customer service easily and were allowed to give feedback about game balance optimization as well as new skin design.

Note that we paid extra effort to rule out other possible reasons which may influence potential findings. A newly designed skin and newly prepared contents were identically provided for all of the participants in the two conditions. The only difference between the two conditions was that participants had the possibility of interacting with the game company.

### 3.4. Procedure

To avoid any potential threat from COVID-19, we conducted our experiment remotely using participants’ mobile phones. Participants were invited to join the Tencent meeting room to control the features of a hypothetical mobile game system and experience the game. When they entered the meeting room, they confirmed voluntary participation, signed the informed consent form, and were introduced to the introductory screen of the latest version of Honor of Kings. Note that participants were unable to control buttons on the game. If they wanted to press a button, for instance, they requested the experimenter to do so. When the experimenter pressed the requested button, they watched the outcome together through a shared screen. Participants were allowed to request that the experimenter press any button on the screen to obtain information about the game balance optimization and the new skin design. After pressing buttons and experiencing the game, they answered several questions and were thanked and debriefed.

### 3.5. Measure

In the survey, we measured psychological ownership and in-app purchasing intention. Psychological ownership was measured by three questions (this is my mobile game, I feel a very high degree of personal ownership for this mobile game, and I sense that this mobile game is our mobile game [18]. In-app purchasing intention was measured by five questions (I intend to continue purchasing in this mobile game, I strongly recommend others to purchase in this mobile game, I find purchasing in this mobile game to be worthwhile, I am likely to purchase in this mobile game in the future, and I plan to spend more on this mobile game [8]. The eight questions were answered using a 5-point Likert scale from 1 (strongly disagree) to 5 (strongly agree). Each questionnaire is included in Appendix A.

### 3.6. Analysis

We analyzed the collected responses using SPSS 26 and PROCESS macro, as proposed by Preacher and Hayes [32] because similarly collected data were handled and analyzed in the same way in the past [33]. To test the two hypotheses, we used independent samples tests and regression analysis. In particular, we used Hayes Model 4 to test whether psychological ownership mediated the relationship between value co-creation and in-app purchasing intention.

### 3.7. Result

#### 3.7.1. Demographic Information of Participants

A total of 86 participants volunteered to participate in this experiment. The basic information of participants is shown as follows (Table 1).

#### 3.7.2. Testing Hypothesis

**Figure 2:**
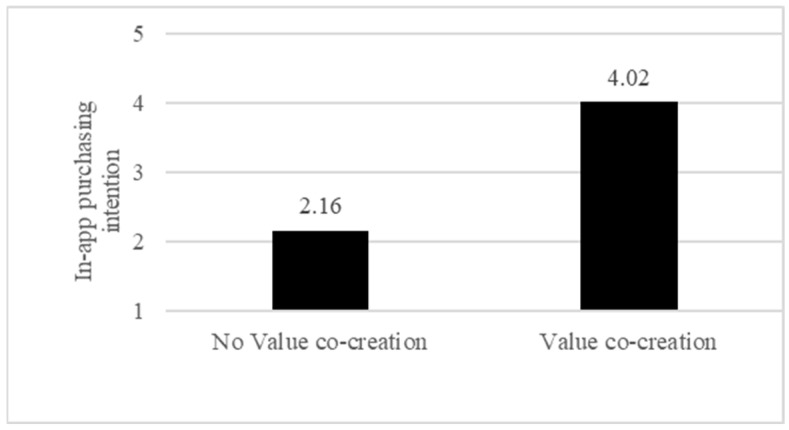
In-app purchasing intention as a function of value co-creation.

First, our independent sample test supported H1, that is, value co-creation increases consumers’ in-app purchasing intention. When participants were allowed to co-create the value in the game, their in-app purchasing intention is greater than when they were not allowed to do so when value co-creation did not exist (M_Value co-creation_ = 4.02 vs. M_No value co-creation_ = 2.16, *t*(84) = 11.27, *p* < 0.001) (Figure 2).

Second, our bootstrapping analysis, as suggested by Preacher and Hayes [32], supported H2, that is, the mediating effect of psychological ownership on the relationship between value co-creation and in-app purchasing intention. We conducted PROCESS macro and followed bias-corrected bootstrapping (CI = 95%) which used 5000 bootstrap samples to test. We chose model 4 in SPSS and analyzed mediating effect by setting value co-creation as an independent variable, psychological ownership as a mediator, and in-app purchasing intention as a dependent variable. The results of the bootstrap test show that the total effect of value co-creation on in-app purchasing intention is 1.866 (95% CI = 1.54~2.20), its direct effect is 0.941 (95% CI = 0.52~1.36), and its indirect effect is 0.926 (95% CI = 0.59~1.31). Since the 95% confidence interval of the indirect effect does not contain 0, results suggest that psychological ownership mediates the relationship between value co-creation and in-app purchasing intention (Table 2).

## 4. Conclusions

### 4.1. Discussion

Our experimental study demonstrates that consumer value co-creation with a mobile game company will increase their in-app purchasing intention (H1). More importantly, psychological ownership played a mediating role between value co-creation and in-app purchasing intention. That is, when consumers co-create value with a mobile game company, they feel that the mobile game is their own mobile game (psychological ownership of the mobile game increases), which leads them to increase their intention for in-app purchasing (H2). Specifically speaking, our findings suggest that when people co-create value with a mobile game company on an imaginary mobile game interface, they are more willing to buy virtual products available in the mobile game. 

Our experimental findings diverge from previous studies in two important ways. Firstly, while earlier research has suggested that consumer behavior is influenced either by the factors of the company, such as interfaces [5], or the factors of the consumers, such as their goals, our study shows that the relationship between the user and the game company plays a decisive role in shaping user behavior, such as their in-app purchasing intentions. Secondly, in addition to the previously identified psychological factors such as achievements, loyalty, addiction, and perception of game items that motivate users to spend money on virtual currency and in-game items [4,8], we have discovered a novel psychological factor that increases their in-app purchasing intentions: users’ psychological ownership driven by their value co-creation activities.

### 4.2. Contributions

Its academic contribution is divided into two parts. First, in this study, we put forward that value co-creation effectively increases in-app purchasing intention. Although in-app purchasing intention was discussed extensively, prior work focuses rather on describing phenomena. Prior studies show that consumers are more likely to purchase in-app items, for instance when they play the game for a long time [34], are satisfied with or loyal to the game [35], or when they are surrounded by other game players [36]. Different from previous research, our research picks up the same topic and addresses it from a different, prescriptive perspective; it aims to provide insights for mobile game marketers who struggle with elevating in-app purchasing intention. Marketers will be able to add features reflecting value co-creation in the future. 

Second, we clarify that consumer value co-creation leads to psychological ownership and greater marketer profits. When consumers establish an emotional attachment to the game company, their purchase behavior inside the game could be promoted. Although prior studies show how psychological ownership affects attitudes, values, and behaviors in general [23], their findings focus on either tangible products or public goods [24]. Our research is the first case to show that psychological ownership activated by briefly experiencing a virtual game could influence consumer behavior toward a non-tangible, virtual entity. 

Its practical contribution is clear and easy to implement. Faced with declining revenue due to increased competition, mobile game companies paid attention to in-app purchases by users. Our study demonstrated that the strategies and interfaces developed by the game company are not the only factors that influence in-app purchasing but also the relationship between the user and the game company. Now, game companies should engage with users more actively and intensively when developing new games and through online gaming communities.

### 4.3. Limitations

This study has several limitations that call for deeper research on the value co-creation of mobile games between consumers and mobile game companies. First, we designed a hypothetical mobile game interface and then invited participants to co-create value with the mobile game company by giving feedback about game balance adjustment and new skin design. However, we noticed that some participants paid little attention to the aesthetic attributes of new skin designs, suggesting that different people give different weights to the evaluation of the same things. For instance, when consumers evaluate products, they consider aesthetic attributes and functional attributes differently [37]. Therefore, in the future, researchers must test the same hypotheses among different groups of people in different types of mobile games.

Second, our participants are adults with mobile game experience, but they might be different in terms of expertise. Research shows that casual game players behave differently from hard-core game players [38]. There is evidence that novices are attracted by aesthetically pleasing products more strongly than experts [39]. Therefore, there is research in which design needs to be taught differently depending on students’ expertise and characteristics [40]. The same rule should be applied to the mobile game context. Different game players exposed to different value co-creation features will result in different attitudes and behaviors in the mobile game context. In the future, researchers should study different players more deeply in terms of value co-creation.

Finally, we exclusively enrolled Chinese participants, given the thriving gaming industry in China and its high rate of game usage [41]. However, we cannot ensure that the generalizability of our findings transfers to countries where mobile gaming is not as prevalent. Hence, we acknowledge this geographic constraint and recommend that future research examine the same hypotheses among diverse populations in other countries [42,43].

## Figures and Tables

**Table 1:** Demographic information of participants.

	*n* (%)	M (SD)
Male	37 (43%)	
Female	49 (57%)	
Age		24.05 (2.86)

**Table 2:** Mediating test of psychological ownership between value co-creation and in-app purchasing intention.

Total Effect of Value Co-Creation on In-App Purchasing Intention					
Effect	SE	*t*	*p*	LLCI	ULCI
1.866	0.165	11.251	0.000	1.536	2.196
The direct effect of value co-creation on in-app purchasing intention					
Effect	SE	*t*	*p*	LLCI	ULCI
0.941	0.213	4.404	0.000	0.516	1.365
The indirect effect of value co-creation on in-app purchasing intention					
	Effect	BootSE	BootLLCI	BootULCI	
PO	0.926	0.184	0.589	1.313	

## Data Availability

The data collected and presented in this study are available upon request.

## Appendix A

Psychological ownership (1 = strongly disagree vs. 5 = strongly agree):

- This is my mobile game.
- I feel a very high degree of personal ownership for this mobile game.
- I sense that this mobile game is our mobile game.

In-app purchasing intention (1 = strongly disagree vs. 5 = strongly agree):

- I intend to continue purchasing in this mobile game.
- I strongly recommend others to purchase in this mobile game.
- I find purchasing in this mobile game to be worthwhile.
- I am likely to frequently purchase from this mobile game in the future.
- I plan to spend more on this mobile game.
